# Resistome and virulome determination in *Helicobacter pylori* using next-generation sequencing with target-enrichment technology

**DOI:** 10.1128/spectrum.03298-24

**Published:** 2025-03-05

**Authors:** Léo Gillet, Lucie Bénéjat, Quentin Jehanne, Pierre-Louis Maunet, Claudie Perreau, Astrid Ducournau, Johanna Aptel, Marine Jauvain, Philippe Lehours

**Affiliations:** 1 CHU de Bordeaux, CNR des Campylobacters et des Hélicobacters, Bordeaux, France; 2 INSERM U1312, UMR BRIC-Team 4, Bordeaux, France; Assistance Publique-Hopitaux de Paris Universite Paris Saclay, Clamart, France

**Keywords:** next-generation sequencing, *H. pylori*, resistome, gastric biopsy, target-enrichment

## Abstract

The identification of *Helicobacter pylori* infection from gastric biopsy samples requires PCR or bacterial cultures. However, it is difficult to culture *H. pylori* because it is a fragile bacterium. Next-generation sequencing (NGS) allows direct assessment of the resistome and virulome. Here we describe a new NGS method for studying the resistome and virulome of *H. pylori* directly from gastric biopsies, based on enrichment analyses and targeted sequencing of *H. pylori* DNA. In all, 19 DNA samples from human gastric biopsies that tested positive for *H. pylori* were analyzed. The Agilent SureSelectXT target-enrichment protocol was used with a custom bait library prior to sequencing using the Agilent MagnisDx NGS Library Prep System. NGS sequencing was performed on the Illumina iSeq 100 sequencer using RNA probes for virulence, resistance, and molecular typing genes. The method yielded significant results with a limit of detection of around 1.8e^5^ CFU per mL *H. pylori*. Mutations in the *23S rDNA* sequence associated with macrolide resistance and in the quinolone resistance-determining region of *gyrase A* associated with levofloxacin resistance were correctly identified. The results of MLST phylogeny analyses performed after target-enrichment were consistent with those obtained via conventional Sanger sequencing. Among the *cagA*-positive isolates, the gene was detected correctly, and the *vacA* genotype was determined. In conclusion, our enrichment method enables rapid assessment of the resistome and virulome of *H. pylori* directly from fresh gastric biopsies.

**IMPORTANCE:**

*Helicobacter pylori*, a bacterium that infects at least 50% of the world population, is often treated by probabilistic antimicrobial therapies due to the lack of antimicrobial resistance data provided by clinical laboratories to clinicians. However, targeted antimicrobial therapies are increasingly recommended to achieve efficient eradication with a limited impact on the gut microbiota and with fewer adverse events for the patient. Recent advancements in next-generation sequencing strategies have opened new opportunities in the diagnosis of *H. pylori* infection. The significance of our research is the development of a novel next-generation sequencing strategy based on target-enrichment. This approach enables the identification of the resistome and the virulome of *H. pylori* directly from gastric biopsies, providing clinicians with a broad overview of therapeutic options.

## INTRODUCTION

*Helicobacter pylori* is a Gram-negative, microaerophilic, coiled, and flagellated bacterium that exclusively infects the human stomach. Its role in gastroduodenal diseases, notably gastric ulcers, was established, and in 1994, the International Agency for Research on Cancer classified the bacterium as a class I carcinogen (1). It is the most common cause of cancers of infectious origin, which makes stomach cancer the sixth most common, and the fourth most deadly, cancer (2). Eradication of the bacterium cures gastritis and may alter the long-term course of complications of the disease (3).

Virulence factors of *H. pylori* enable its survival, colonization, and persistence in the acidic environment of the stomach. The *cagA* gene is part of a pathogenicity island (*cagPAI*) that codes for about 30 proteins that make up the type IV secretion system (T4SS). CagA is an oncoprotein and the major virulence factor that drives inflammation and proliferation and inhibits cell polarity and apoptosis. Its biological activity is linked to the presence of repeated C-terminal phosphorylation motifs (4). The *vacA* gene, which encodes the VacA cytotoxin, is present in all *H. pylori* strains but is not always expressed (5) due to genetic polymorphisms in the N-terminal signal sequences (s1 or s2), the middle region (m1 or m2), and the intermediate region (i1 or i2). The s1/m1 polymorphism is correlated with elevated risks of gastric ulcers and cancer (6).

Invasive and noninvasive diagnostic methods are available for the detection of *H. pylori*, depending on whether gastric biopsy is required (7). Noninvasive methods include the urea breath test, serology, and stool tests for *H. pylori* antigens. Invasive methods are the most sensitive and specific (8) and require the collection of gastric biopsies during upper digestive endoscopy. The biopsies can be sent to a pathology laboratory to analyze tissue lesions and detection of *H. pylori*. The disadvantage of histology is that, unlike noninvasive methods, it does not allow investigation of antimicrobial resistance.

The bacteriological examination of biopsies by culture on agar media has the highest specificity and enables antimicrobial susceptibility testing (AST) (8). However, not all laboratories can cultivate *H. pylori* because it grows slowly and its culture is problematic, requiring expertise. After extraction of DNA from a biopsy, molecular detection of the bacterium can be performed using commercially available, real-time PCR kits that can detect macrolide resistance more rapidly and more sensitively than culture (9, 10).

Given the increasing resistance of *H. pylori* to antimicrobials, *in vitro* AST prior to treatment is needed for antibiotic susceptibility-guided eradication therapy (7). If AST is not performed, empirical treatment will be initiated (11). Unfortunately, even in specialized laboratories, PCR-positive biopsies for *H. pylori* can remain negative in culture.

New methods using next-generation sequencing (NGS) have been developed to alleviate this problem. In 2018, Lauener et al. showed that it is possible to detect mutations responsible for resistance to clarithromycin, levofloxacin, rifampicin, and tetracycline using bacterial DNA and NGS, with more than 99% congruence with phenotypic results (12). An NGS method, commercially available only in the United States, applied directly to fresh gastric biopsies, paraffin-embedded biopsies, or stool samples enables assessment of the *H. pylori* resistome (13) (14). This important technological advance allows the implementation of targeted therapies based on molecular rather than phenotypic data.

Other NGS strategies use target-enrichment, which allows larger numbers of genes to be sequenced. The target-enrichment strategy has been used for whole-human-exome sequencing as well as research on human cancers and genetic diseases, and its flexibility allows the design of probes for any target (15). This technique involves the development of specific DNA or RNA probes that bind to the desired regions by complementarity, enabling specific sequencing. It can be used to isolate and specifically amplify regions of the genome of a pathogen in biological samples, which can be subjected to high-quality sequencing. It has been evaluated and validated for *Chlamydia trachomatis* (16) (17) and *Mycobacterium tuberculosis* (18).

In this study, we developed a target-enrichment technique to evaluate the resistome and virulome of *H. pylori* directly from gastric biopsies. The results were compared to those obtained using conventional PCR and Sanger sequencing methods. We validated the method for *H. pylori* diagnosis as it provides reliable results.

## MATERIALS AND METHODS

### Samples

In all, 19 gastric biopsies received at the National Reference Center for Campylobacter and Helicobacter (NRCCH) (http://www.cnrch.fr) between April and December 2022 were included in the study. All of the biopsies were from patients living in France, with a mean age of 49.5 ± 18 years and a sex ratio of approximately 0.46 (Table 1). The biopsies were confirmed to be positive for *H. pylori* in culture and via real-time PCR.

**TABLE 1:** Description of cases

Sample	Sex	Age	Previous eradication	Pathology, symptoms	*H. pylori* ct	*23S rDNA* genotype determined by RT-PCR^*a*^	AST resistance marker^*b*,*c*^
1	M	60	No	Gastritis	26.6	A2142G or A2143G	Cla
2	M	84	Yes	MALT lymphoma	25.7	A2142G or A2143G	Cla, Lev
3	F	71	No	Gastritis	19.6	WT + A2142G or A2143G	Cla
4	F	34	No data	No data	19.5	WT	Lev
5	F	41	No	Other inflammatory bowel diseases (IBD)	19.5	WT	Cla
6	M	34	No	Gastritis	22.8	A2142G or A2143G	Cla
7	F	39	No	Gastroesophageal reflux disease (GERD)	23.4	WT	None
8	F	22	No	Gastritis	25.9	A2142C	Cla, Lev
9	F	31	No	Emesis	19.5	WT	None
10	F	49	Yes	Gastritis	18.3	WT + A2142G or A2143G	Cla
11	F	27	No	Gastritis	19.3	WT	None
12	F	62	No	Epigastralgia	19.8	A2142G or A2143G	Cla, Lev
13	M	41	No	Gastritis	19.1	WT	None
14	F	58	No	Dyspepsia	20.3	WT	None
15	F	32	Yes	Gastritis	23.3	WT + A2142G or A2143G	Cla
16	F	71	Yes	Gastritis	23.3	WT	Lev
17	F	54	No	Epigastralgia	20.1	WT + A2142G or A2143G	Cla
18	M	72	No	Positive serology	25	A2142G or A2143G	Cla, Lev
19	M	59	No	Gastritis	27.5	A2142G or A2143G	Cla, Lev

^*a*^ WT, wild type.

^*b*^ AST, antimicrobial susceptibility testing.

^*c*^ Cla, clarithromycin; Lev, levofloxacin; Rif, rifampicin; Tet, tetracycline.

Upon receipt, according to our routine protocol, the biopsies were ground in 500 µL nutrient broth and stored at –80°C. A portion of the suspension was treated with 20 µL proteinase K (Roche Diagnostica, Meylan, France) in 180 µL ATL Qiagen buffer at 56°C for 3 h, and 200 µL was used on ground biopsies before DNA extraction on a MagNA Pure 96 system (Roche Diagnostics) and PCR on Eurogentec strips (Liège, Belgium) using an LC480 (Roche Diagnostics) (19). The threshold cycle values (Ct) determined via real-time PCR ranged from 18.3 to 27.5 (Table 1).

Bacterial culture was performed in parallel according to internal laboratory procedures (20). The strains were cultured on in-house blood agar containing antimicrobials as described previously (20). The media were incubated in a microaerobic atmosphere in a special workstation (Baker Ruskinn, Concept Ruskinn, Bridget, UK) at 37°C for 10 days. After 2 days, the plates were observed daily, and colonies were tested for oxidase, catalase, and urease and underwent morphological observation. AST was performed on Mueller–Hinton agar containing 10% horse blood, using Etest strips (bioMérieux) for clarithromycin, levofloxacin, rifampicin, and tetracycline. The cutoff values were those recommended by the French Microbiology Society Antibiogram Committee (CASFM) (CA-SFM/EUCAST: Société Française de Microbiologie Ed; 2024: pp. 1–177). For each strain, the MICs of clarithromycin, levofloxacin, rifampicin, and tetracycline were measured by two independent readers. Quality control was performed using *H. pylori* strain CCUG 17874.

### End-point PCR

DNA was extracted from gastric biopsies to determine the *vacA* and *cagA* genotypes via PCR, to detect resistance markers (quinolone resistance-determining region [QRDR] of *gyrA* for levofloxacin resistance), and to extract genes for MLST typing, using primers described previously (https://pubmlst.org/organisms/helicobacter-pylori/primers) (Table S1). The reaction consisted of 5 µL 5 × PCR buffer (Promega, Madison, WI), 2.5 µL MgCl_2_ (Promega), 0.5 µL dNTPs (Promega), 0.125 µL GoTaq G2 Hot-Start Polymerase (Promega), 1 µL each primer at 10 µM (Eurofins Genomics, Ebersberg, Germany), and 5 µL extracted DNA in a final volume of 25 µL. Amplification parameters consisted of 1 cycle at 95°C for 5 min, followed by 40 cycles at 95°C for 30 s, 54°C for 30 s, and 72°C for 90 s, and finally 1 cycle at 72°C for 5 min. The amplified fragments were resolved on 2% agarose gels containing green Midori Pre-stain (Nippon Genetics Europe, Düren, Germany). The PCR products of *gyrA* and MLST typing genes were sequenced by Eurofins Genomic. The sequences were analyzed using the NCBI Nucleotide BLAST (21) and aligned with MultAlin (22) if necessary.

### Probe design for target-enrichment

An RNA probe library of 13,245 120-mer probes with a total size of 108 kbp was designed by Agilent using the selected regions of interest of the *H. pylori* reference strain J99 (23) and the reference strain B38 (24). The use of these genotypes resulted in a library containing probes that can hybridize to all s/i/m *vacA* genotypes. To enrich the library with probes specific for *cagA* EPIYA phosphorylation motifs, we used reference strains with multiple repeats of the C motif (involved in host interaction), NCTC 11637, and MT5114 (25) (which has the D motif common in Asian *H. pylori* strains), F32, and F17 (26, 27, 28). The probe library enabled the detection of mutations associated with resistance to clarithromycin (*23S rDNA*), levofloxacin (*gyrA*), rifampicin (*rpoB*), and tetracycline (*16S rDNA*) (Table 2). All of the regions of interest typically used in *H. pylori* MLST were targeted (29).

**TABLE 2:** Mutations associated with phenotypic resistance to antibiotics

Gene	Mutations	Associated resistance
*16S rDNA*	AGA_926-928_TTC, AGA_926-928_ATC, AGA_926-928_TTA, AGA_926-928_TGC, AGA_926-928_AGC, AGA_926-928_ATA	Tetracycline
*23S rDNA*	A2142C, A2142G, A2142T, A2143C, A2143G	Clarithromycin
QRDR *(gyrA)*	D86N, N87K, N87I, N87Y, A88P, A88V, D91G, D91N, D91Y	Levofloxacin
*rpoB*	L525I, L525P, S526T, Q527H, Q527K, Q527R, F528E, D530A, D530E, D530G, D530N, D530V, T539A, H540C, H540N, H540Y, S545L, L547F, I586N, I586L, T588NL	Rifamycin

### Automated capture-based library preparation

Most steps of the SureSelectXT HS2 workflow were automated using the Agilent MagnisDx library preparation platform from enzymatic fragmentation to the capture of hybridized libraries. The samples were diluted to 50 to 100 ng total DNA in a 14 µL input volume. Following Agilent’s recommendation, the enzymatic fragmentation duration was set to 15 min and the read-length was two times 150 bp. After multiple calibration tests (data not shown), sequencing quality was maximized by setting 20 pre-capture and 24 post-capture PCR cycles.

### NGS short-read sequencing

The fragment size distribution and molarity of the libraries were evaluated using the Agilent TapeStation 4150 System and High-Sensitivity D1000 assay. Each sample (2 µL) was diluted 1:10 and mixed with 2 µL High-Sensitivity D1000 Sample Buffer. The libraries were pooled at an equimolar concentration of 0.125 nM. Twenty microliters was harvested for multiplex sequencing on the Illumina iSeq 100 Sequencer at the NRCCH.

### Bioinformatics workflow

Raw reads were mapped to the host genome using Bowtie2 v0.7.17 and the *Homo sapiens* GRCh38 reference genome. Unmapped reads were trimmed and cleaned using fastp v0.23.4 (30). A deduplication step, taking advantage of the duplex metabarcoding of the inserts, was used to generate consensus sequences and reduce false-positive calls. Reads specific to *H. pylori* were selected using Kraken2 v1.2.2 (31), a taxonomic classification system that employs exact k-mer matches, with the PlusPF database, which contains RefSeq bacterial, plasmid, viral, human, protozoal, and fungal sequences. The reads were mapped onto *H. pylori* reference genome J99 (assembly ASM98269v1) (23) using Bowtie2 (32), and the consensus sequences of the targeted regions were extracted using Freebayes v1.3.6 (33) and bcftools v1.1.19 (34). A homemade suite of tools was used with those consensus sequences to detect genomic markers and assess the resistome. In parallel, a *de novo* assembly was generated using the NCBI SAUTE v2.5.1 (35), to generate complete sequences of *cagA* and *vacA*. MLST sequences were concatenated and used as input for STRUCTURE v2.3.4 (36), attributing each sample to a range of annotated populations. They were compared to the automatically updated and annotated database of allele sequences, available on PubMLST (37). Phylogenetic trees were generated using RAxML-NG v1.2.2 (38) using the GTR + Gamma model. A set of homemade scripts was developed to compile the results and generate PDF and HTML reports, as well as a circular genome representation, using the Circos graphic library v0.69–9 (39).

### Limit of detection

One *H*. *pylori*-negative gastric biopsy sample (by PCR and culture) was ground, homogenized, and spiked with *H. pylori* CCUG 17874 (40, 41) at 1.8 to 1.8e^7^ CFU per mL and enzymatically digested overnight at 56°C in 180 µL ATL buffer (Qiagen) and 20 µL proteinase K (Roche Diagnostics). The libraries were prepared three times and sequenced using the same protocol as the clinical samples to identify cutoff Ct and bacterial charge values that ensured a good sequencing quality and accurate evaluation of resistome and virulome.

### Serial dilution of mixed samples

To evaluate the ability of our technique to detect mixed infection with susceptible (S) and resistant (R) strains, artificial mixes of the clarithromycin- and levofloxacin-susceptible *H. pylori* reference strain CCUG 17874 with clarithromycin- and levofloxacin-resistant clinical isolate (AST data + PCR data) were prepared. Each strain was suspended at a turbidity of 1 McFarland in brain–heart infusion broth and transferred to 1:1 (500 µL each strain), 1:5 (800 µL the susceptible strain, 200 µL the resistant strain), 1:10 (900 µL S, 100 µL R), and 1:100 (990 µL S, 10 µL R) mixtures in duplicate. The mixtures were centrifuged for 10 min at 13,000 RPM before extraction, following the same protocol as that used for the spiked samples. Mutations in *23S rDNA* were detected using real-time PCR, and mutations in the QRDR region of *gyrA* were detected via PCR and Sanger sequencing, using the primer pairs given in Table S1.

### Whole-genome sequencing of *cagA*-positive samples

*cagA*-positive strains were sent for whole-genome sequencing at the GenoBioMICS sequencing platform (CHU Henri Mondor, Créteil, France) to determine the EPIYA motifs of CagA. Total DNA was extracted using mechanical (bead-beating), enzymatic, and chemical methods on the Revvity Chemagic Prime instrument. Libraries were prepared using the Illumina DNA Prep Kit on the Tecan DreamPrep NGS system and sequenced using an Illumina NovaSeq 6000 sequencer. Bioinformatic analysis was performed using the pipeline described above, with the exception of the genome assembly step, which was conducted using NCBI SKESA v2.5.1 (35). A supplementary assembly annotation step was added to extract *cagA* sequences using Prokka v1.14.5 (42).

## RESULTS

### Sensitivity and limit of detection

**Fig 1:**
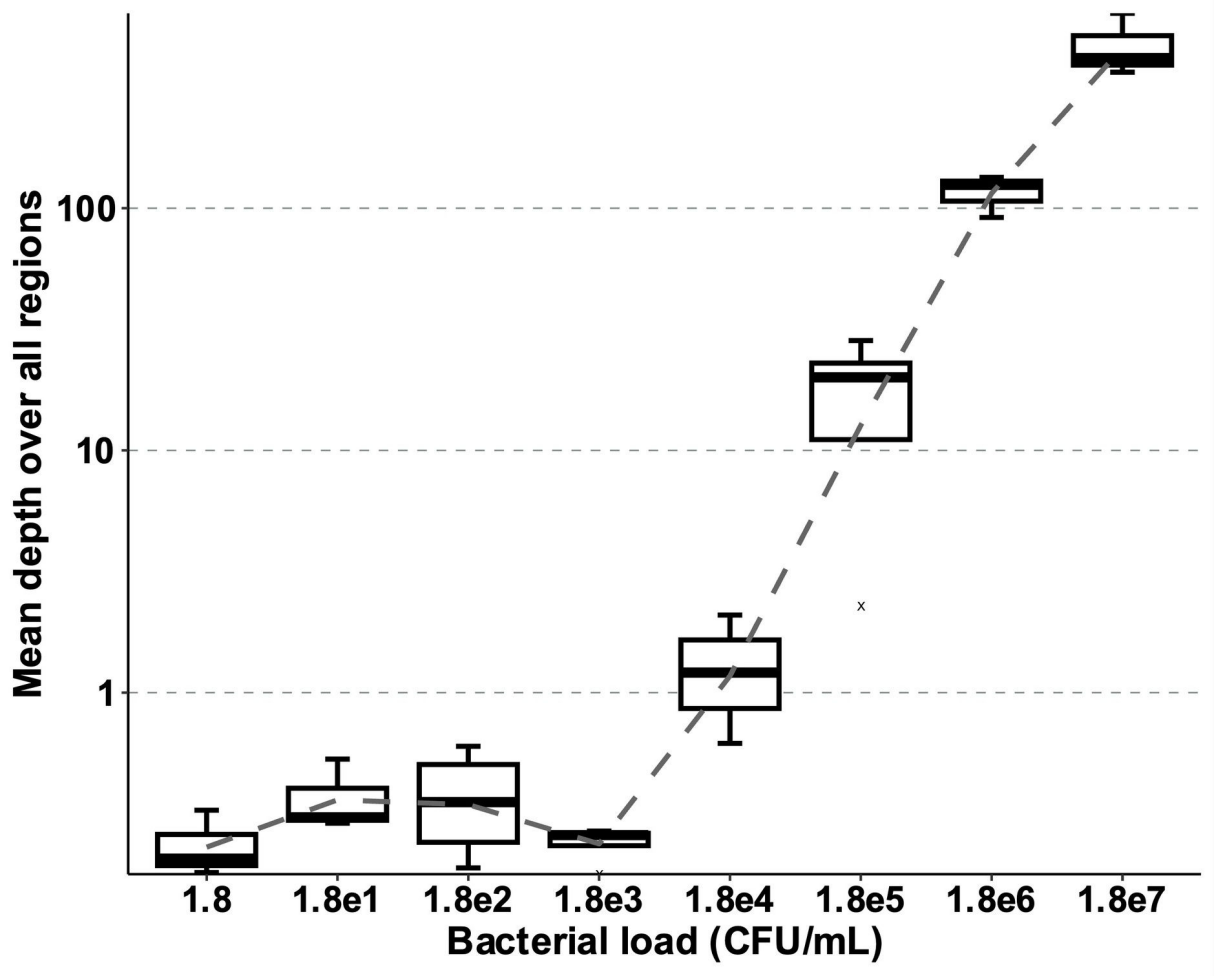
Limit of detection in serial dilutions of gastric biopsies spiked with the *H. pylori* reference strain CCUG 17874. Values are average mean depths overall targets, with a threshold of 10 reads per base.

**Fig 2:**
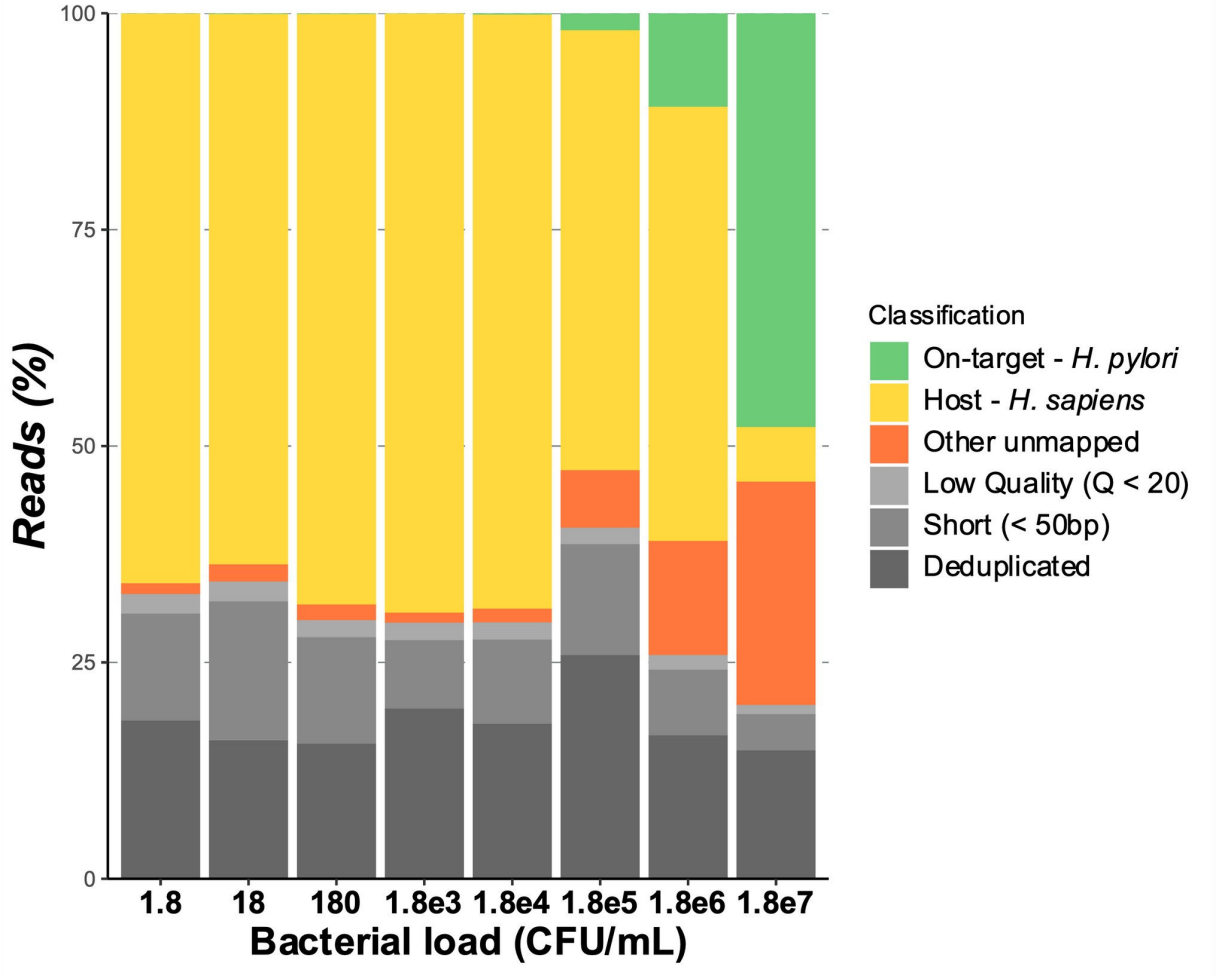
Classification of sequencing reads depending on the bacterial load (CFU per mL). Although other *unmapped* reads may correspond to other sequenced organisms either by accidental contamination or capture of conserved genomic sequences, metagenomic classification of raw reads using Kraken2 did not reveal the presence of particular species but detected a handful of species at low levels and unclassifiable reads.

The number of reads attributed to *H. pylori* and the coverage of regions at minimum depth (5× +metabarcoding consensus) are shown in Fig. 1. The percentage of reads attributed to *H. pylori* was <10% for DNA extracted from gastric biopsies spiked with <1.8e^5^ UFC per mL. As expected, the reduction in bacterial load was accompanied by an increase in the percentage of human reads Fig. 2. The sequencing data of the spiked gastric biopsies and the samples are listed in Table S2.

### Limits of detection for *23S rDNA* and *gyrA* mutations in mixed populations

To assess the sensitivity of the technique for detecting antibiotic-resistant mixed populations, a series of mixtures of different *H. pylori* strains were prepared (one sensitive and the other resistant to clarithromycin and levofloxacin) and sequenced using the same library preparation protocol. At a 1:1 susceptible:resistant strain ratio, approximately half of the sequencing reads at position A2143 of the *23S rDNA* and G271 of *gyrA* had SNPs associated with resistance to clarithromycin (A2143G) and levofloxacin (D91Y), respectively. The other sequencing reads corresponded to wild-type genotypes.

As the concentration of resistant bacteria in the mixtures decreased, the proportion of mutated alleles decreased. Mutated alleles were detected at a 1:10 susceptible:resistant strain ratio. At a 1:100 ratio, few reads had mutations associated with resistance to both antimicrobials; these were not detected using our technique (Table 3).

**TABLE 3:** Percentages of mutant alleles in mixtures of susceptible and resistant strains

Theoretical ratio (percentage of resistant individuals)	*23S* A2143G allele percentage	Base depth at position	*gyrA* D91Y allele percentage	Base depth at position
1:1 (50%)	49%	318	49%	582
1:5 (20%)	13%	264	13%	635
1:10 (10%)	5%	177	6%	536
1:100 (1%)	<0.01%	445	<0.01%	517

### Detection of antimicrobial resistance markers in clinical isolates

To evaluate our method using clinical specimens, 19 samples were tested. All samples tested positive via real-time PCR for *H. pylori* with Ct values < 27 (Table 1), consistent with the sensitivity mentioned above. Mutations in *23S rDNA* identified via target-enrichment were compared to those determined through real-time PCR. The correlation was perfect in 17 of the 19 samples tested, and all genotypes were correctly identified (Table 4). The main mutation identified was A2143G. Base depth was insufficient for samples 2 and 7. A mixed population of wild-type and mutant *H. pylori* was detected in samples 10, 12, 15, and 17, with the mutant population representing 10% to 70% of the called bases. Surprisingly, only A2143G was detected in sample 3, and the double population originally detected via real-time PCR was absent. The presence of a mixed population (S + R) was confirmed *in vitro* via AST using Etest on the strain isolated from this sample and by genome sequencing (WT +A2143G 60%) (data not shown). In sample 12, the mutations A2142G (85% of bases called) and A2143G (13% of bases called) were identified, indicating a mixture of clarithromycin-resistant *H. pylori* populations.

**TABLE 4:** *23S rDNA* mutations detected via real-time PCR and target-enrichment analysis^*a*^

Sample	Observed mutation by RT-PCR	Observed mutation by target-enrichment
1	A2142G or A2143G	A2143G
2	A2142G or A2143G	Insufficient base depth
3	WT + A2142G or A2143G	A2143G
4	WT	WT
5	WT	WT
6	A2142G or A2143G	A2143G
7	WT	Insufficient base depth
8	A2142C	A2142C
9	WT	WT
10	WT +A2142G or A2143G	WT + A2143G (10% of called bases)
11	WT	WT
12	A2142G or A2143G	A2142G (85%), A2143G (13%)
13	WT	WT
14	WT	WT
15	WT + A2142G or A2143G	WT + A2143G (70%)
16	WT	WT
17	WT + A2142G or A2143G	WT + A2143G (32%)
18	A2142G or A2143G	A2143G
19	A2142G or A2143G	A2143G

^*a*^ For samples with mixed populations, such as samples 10, 12, 15, and 17, the percentages of variants are shown.

The mutations in the QRDR region of *gyrA* identified via target-enrichment were compared to those identified through endpoint PCR and Sanger sequencing. Excluding sample 11 with insufficient base depth, the correlation was perfect in all the remaining cases (Table 5), and all of the mutations were correctly identified. The mutations associated with levofloxacin resistance were D91N (two samples), N87K (four samples), and N87I (one sample). In samples 5 and 19, 53% and 25% of the called bases were identified, respectively. This indicates the presence of a mixture of wild-type and mutant *H. pylori*. No evidence of mixed populations was found in these samples via *in vitro* AST using Etest. No mutation of *gyrA* associated with levofloxacin resistance was missed in these two samples using target-enrichment.

**TABLE 5:** *gyrA* mutations in gastric samples detected via end-point PCR and Sanger sequencing and target-enrichment analysis^*a*^

Sample	Observed mutation by PCR & Sanger sequencing	Observed mutation by target-enrichment
1	WT	WT
2	D91N	D91N
3	WT	WT
4	N87K	N87K
5	N87K	WT + N87K (53%)
6	WT	WT
7	WT	WT
8	N87K	N87K
9	WT	WT
10	WT	WT
11	WT	Insufficient base depth
12	N87I	N87I
13	WT	WT
14	WT	WT
15	WT	WT
16	N87K	N87K
17	WT	WT
18	D91N	D91N
19	N87K	WT + N87K (25%)

^*a*^ For samples with mixed populations, such as samples 5 and 19, the percentages of variants are shown.

No resistance marker for tetracycline and rifampicin was found in the 19 samples (data not shown). This was expected because no *H. pylori* isolates resistant to these drugs were included in this study. However, the average 5× coverages over the sites searched for potential antimicrobial markers in the 19 samples for *16S rDNA* and *rpoB* were 94.7% (± 23%) and 84.7% (± 36%), respectively (Table S3). This indicates perfect sequencing of genes involved in tetracycline and rifampicin resistance. The exceptions were samples 2, 7, 8, and 16, which had poor sequencing quality over the targeted regions.

### MLST of clinical samples

Geographic attribution of samples was computed from the concatenated sequences of the *H. pylori* typing scheme using STRUCTURE and a database of annotated sequences from PubMLST. The results of end-point PCR followed by Sanger sequencing were compared to those of target-enrichment (Table 6). The correlation between the two approaches was perfect. Most of the biopsies (14 of 19, 73.7%) contained hpEurope *H. pylori*. Three biopsies contained hpEurope and hpAfrica1 hybrids (3 of 19, 15.8%) and *H. pylori* strains with hpAfrica1 represented 25% to 50% of the profile. The two other cases (2 of 19, 10.5%) were attributed to hpAfrica1.

**TABLE 6:** MLST typing and hpGroup determined via end-point PCR plus Sanger sequencing and target-enrichment analysis using STRUCTURE

Sample	Assigned geographic profile using Sanger sequencing	Assigned geographic profile using target-enrichment
1	*hpEurope* (65%), *hpAfrica1* (35%)	*hpEurope* (70%), *hpAfrica1* (30%)
2	*hpEurope*	*hpEurope*
3	*hpEurope*	*hpEurope*
4	*hpEurope*	*hpEurope*
5	*hpAfrica1*	*hpEurope*
6	*hpEurope*	*hpEurope*
7	*hpEurope*	*hpEurope*
8	*hpEurope*	*hpEurope*
9	*hpAfrica1*	*hpAfrica1*
10	*hpEurope* (50%), *hpAfrica1* (50%)	*hpEurope* (50%), *hpAfrica1* (50%)
11	*hpEurope*	*hpEurope*
12	*hpAfrica1*	*hpAfrica1*
13	*hpAfrica1*	*hpEurope* (75%), *hpAfrica1* (25%)
14	*hpEurope*	*hpEurope*
15	*hpEurope*	*hpEurope*
16	*hpEurope*	*hpEurope*
17	*hpEurope*	*hpEurope*
18	*hpEurope*	*hpEurope*
19	*hpEurope*	*hpEurope*

### Virulence typing

There was no discrepancy in the detection of *cagA* via target-enrichment compared to end-point PCR (Table 7). The *cagA* sequence assembly was of insufficient length to detect all EPIYA phosphorylation motifs in samples 1 and 16, and discrepancies in the number of EPIYA motifs were found for samples 11 and 12 (1C versus 2C motifs).

**TABLE 7:** Detection of the *cagA* and *vacA* genotypes via target-enrichment analysis

Sample	*cagA* presence determined by PCR on strains and phosphorylation motifs by WGS	*cagA* presence and phosphorylation motifs determined by target-enrichment	*vacA* genotypes using Sanger sequencing	*vacA* genotypes using target-enrichment
1	Pos,^*a*^ ABC	Pos, no motif	s2/i2/m2	s2/i2/m2
2	Neg^*b*^	Neg	s2/i2/m2	Insufficient quality
3	Neg	Neg	s2/i2/m2	s2/i2/m2
4	Neg	Neg	s2/i2/m2	s2/i2/m2
5	Pos, ABC	Pos, ABC	s1/i1/m1	s1/i1/m1
6	Neg	Neg	s2/i2/m2	s2/i2/m2
7	Neg	Neg	s2/i2/m2	Insufficient quality
8	Neg	Neg	s1/i2/m2	m2
9	Neg	Neg	s2/i2/m2	s2/i2/m2
10	Pos, ABC	Pos, ABC	s1/i1/m2	s1/i1/m2
11	Pos, BC	Pos, BCC	s1/i2/m2	s2/i2/m2
12	Pos, ABC	Pos, ABCC	s1/i1/m1	s1/i1/m1
13	Pos, ABC	Pos, ABC	s1/i1/m1	s1/i1/m1
14	Neg	Neg	s2/i2/m2	s2/i2/m2
15	Neg	Neg	s2/i2/m2	s2/i2/m2
16	Pos, ABC	Pos, no motif	s1/i1/m1	s1/i1/m1
17	Pos, ABC	Pos, ABC	s1/i1/m2	s1/i1/m2
18	Neg	Neg	s2/i2/m2	s2/i2/m2
19	Neg	Neg	s2/i2/m2	Insufficient quality

^*a*^ Pos: positive.

^*b*^ Neg: negative.

For *vacA* genotypes, the correlation was perfect for 15 of the 19 samples. Only the m2 genotype was identified in sample 8. No prediction was made for samples 2, 7, and 19 because of insufficient assembly length.

## DISCUSSION

Our NGS approach using target-enrichment technology yielded reliable results for the *H. pylori* resistome and virulome of gastric biopsy specimens.

The use of NGS with gastric biopsies has previously been evaluated (13, 14). However, using classical library preparation approaches, the first barrier to sufficient sequencing quality is the extraction of enough good-quality DNA suitable for NGS. Our strategy requires 50 to 100 ng of total DNA extracted from a biopsy sample, which is feasible in routine practice. The sensitivity of the technique limits its use to biopsies containing >10^4^ CFU/mL, which corresponds to a Ct value of 27 in real-time PCR routinely used by the NRCCH (19). This detection limit would enable the characterization of approximately 68% of biopsies received annually by our reference center that are PCR-positive but culture-negative (data not shown). A large amount of human DNA is present in gastric biopsies and is amplified with *H. pylori* DNA. Therefore, depletion of human DNA using techniques such as nuclease digestion (43, 44) or methylation-based separation (45) prior to *H. pylori* capture could optimize the amplification of bacterial DNA.

In 2014, Christiansen et al. published a similar technique using the same preparation protocol and probes targeting the whole genome of *Chlamydia trachomatis*, the culture of which is difficult. The detection sensitivity was 10-fold higher than the culture of urine and vaginal swabs (16, 17). Moreover, adding multiple reference genomes of *C. trachomatis* facilitated the capture of more diverse strains (46), prompting us to add probes that would ensure the detection of *cagA* genotypes found in Asian strains. Application of this technique to *Mycobacterium tuberculosis* enables the sequencing of strains directly from sputum samples, with accuracy sufficient to identify heterozygous positions at up to a 95:5 ratio and mixed infections at up to a 90:10 ratio.

Our results are in line with these previous studies because our technique detected a mutation in a mixture in which 10% of the population was resistant to clarithromycin. This is particularly useful for diagnosis prior to targeted eradication therapy. The ability to detect mixed populations of resistant and susceptible *H. pylori* is of particular interest because of its ability to rapidly acquire resistance to antimicrobials due to its high point-mutation frequency (47). This is also an advantage compared to conventional AST or Sanger sequencing techniques, which are less efficient at detecting mixed populations. NGS approaches can provide important information for clinicians for targeted eradication strategies using tailored treatment.

The results of resistome determination via target-enrichment analysis were consistent with those of Sanger sequencing and with the resistance phenotypes obtained *in vitro*. The technique was particularly reliable for markers of resistance to clarithromycin, levofloxacin, rifampicin, and tetracycline. The major mutations detected by Sanger and target-enrichment sequencing were A2143G in *23S rDNA* (associated with clarithromycin resistance) and N87K (associated with levofloxacin resistance), as described by others (48). The absence of resistance to rifampicin and tetracycline is expected, given the low prevalence of resistance to these two antibiotics in *H. pylori* in France (NRCCH annual report; https://www.cnrch.fr/).

Our pipeline database was based on the major mutations in *23S rDNA*, *gyrA*, *rpoB*, and *16S rDNA* associated with clarithromycin, levofloxacin, rifabutin, and tetracycline resistance, respectively. It was also enriched by *rpoB* mutations in French rifampicin-resistant clinical isolates collected over the past 8 years by our reference center. The database was created in collaboration with the *Helicobacter pylori* Genome Project (https://dceg.cancer.gov/research/how-we-study/genomic-studies/h-pylori-genome-project), a global multidisciplinary consortium to study the population structure, drug resistance, and pathogenesis of *H. pylori* (49). Therefore, we believe that our database enables reliable detection of the resistome of *H. pylori* for clarithromycin, levofloxacin, rifabutin, and tetracycline, and at a larger scale than described previously (13, 14).

The bait design involved mutations in *rdxA*, *frxA*, and *fdxB* (associated with metronidazole resistance (50, 51) as well as *pbp1* (amoxicillin resistance [52]). Nevertheless, in the absence of proven associations with antimicrobial resistance, we do not present the results for these genes. Our strategy and pipeline can be applied upon publication of mutations or genes associated with resistance to these antimicrobials and the baits will be adapted if necessary.

The lack of predictions for amoxicillin and metronidazole, unlike other studies (13, 14), is not a major problem for clinicians because resistance in these is rare (amoxicillin) or is counterbalanced by an increased dose (amoxicillin can be adapted to body weight) and/or duration of eradication treatment (metronidazole) (53).

Nevertheless, our target-enrichment approach to the virulome needs to be modified. Although *cagA* detection was good, improvements are planned to increase the sensitivity of the technique for detecting CagA phosphorylation motifs. We had to sequence all *cagA*-positive isolates because the multiplex PCR of Argent et al. (54) provided less reliable results (data not shown) than whole-genome sequencing. Baits designed for the capture of *htrA*, a major virulence factor and an essential gene for the survival of *H. pylori* (55–57), could be added to the library to provide further insight into the virulence profile. Although we were able to detect most *vacA* genotypes, improvement is needed because of the high diversity of this gene. The design of new probes for less well-covered areas is therefore needed. Nevertheless, the evaluation of the virulome is of lesser clinical value than the assessment of the resistome. In addition, no consensus guidelines suggest that antibiotic therapy be adjusted based on the virulome. However, determination of the virulome, in particular *cagA* (and the hypervirulent *htrA* genotype), may be of clinical relevance to *H. pylori* infections in patients with gastric adenocarcinoma, a first-degree family history of gastric cancer, or advanced preneoplastic lesions.

MLST classification of the infecting strain can be seen as “icing on the cake.” The majority of patients in this study were infected with European or African strains, in proportions usually observed in the French population. The correlation between host genotype and infecting strain has been proposed by De Sablet as being, in the event of discordance, a negative factor in disease progression (58). It would be interesting to correlate the *cagA* genotype, in particular the presence of an Asian motif, with the MLST profile. Asian strains are more virulent than others (because of the D motif) and are associated with a higher frequency of severe disease.

Our results lead to an important question: where should the target-enrichment strategy for the routine diagnosis of *H. pylori* infection be placed? In our laboratory, access to culture is not a problem, and its performance is good (up to 10%–15% false-negative results); therefore, the target-enrichment strategy could be reserved for PCR-positive but culture-negative biopsies from patients for whom eradication is expected to be of marked clinical benefit (those with ulcers, preneoplastic lesions, cancer, or familial history of cancer). This would provide information important for selecting the optimum *H. pylori* eradication strategy. For users who want to bypass *H. pylori* culture, the implementation of our method would be feasible using Agilent’s MagnisDx NGS Library Prep System.

In conclusion, we describe a high-performance NGS approach for investigating the resistome and virulome of *H. pylori* from gastric biopsy samples.

## Data Availability

Raw sequencing data generated using the target-enrichment library preparation method as well as whole-genome sequencing data of *cagA*-positive samples are available in the NCBI Sequence Read Archive under BioProject number PRJNA1149679.

## AFTER EPUB

*[This article was published on 5 March 2025 with errors in the byline. The byline was corrected in the current version, posted on 11 March 2025.]*

## SUPPLEMENTAL MATERIAL

Table S1 spectrum.03298-24-s0001.docx PCR primers used as controls.

Table S2 spectrum.03298-24-s0002.xlsx Mean depth and coverage over antimicrobial-resistance marker sites.

Table S3 spectrum.03298-24-s0003.xlsx Fivefold coverage over sites searched for potential antimicrobial markers for 16S rDNA, 23S rDNA, gyrA, and rpob.

ASM does not own the copyrights to Supplemental Material that may be linked to, or accessed through, an article. The authors have granted ASM a non-exclusive, world-wide license to publish the Supplemental Material files. Please contact the corresponding author directly for reuse.
