# Supplementary material for: Resistome and virulome determination in Helicobacter pylori using next-generation sequencing with target-enrichment technology
Source: Microbiol Spectr. 2025 Mar 5;13(4):e03298-24. doi: 10.1128/spectrum.03298-24 (PMC11960115; doi:10.1128/spectrum.03298-24)
Supplement: Table S1 — PCR primers used as controls. [file spectrum.03298-24-s0001.docx]

**Suppl Table 1.** PCR primers used as controls.

| Gene | Purpose | Primers | Sequence |
| --- | --- | --- | --- |
| *vacA* | Virulence | VA1F | ATGGAAATACAACAAACACAC |
|  |  | VA1R | CTGCTTGAATGCGCCAAAC |
|  |  | VAGF | CAATCTGTCCAATCAAGCGAG |
|  |  | VAGR | GCGTCAAAATAATTCCAAGG |
|  |  | VacF1 | GTTGGGATTGGGGGAATGCCG |
|  |  | C1R | TTAATTTAACGCTGTTTGAAG |
|  |  | C2R | GATCAACGCTCTGATTTGA |
| *cagA* | Virulence | CAG-A1 | CCATGAATTTTTGATCCGTTCGG |
|  |  | CAG-A2 | GATAACAGGCAAGCTTTTGAGGGA |
|  |  | CAG-A3 | ATGGGGAGTCATGATGGCATAGAACC |
|  |  | CAG-A4 | ATTAGGCAAATTAAAGACAGCCACC |
| *gyrA* | Levofloxacin sensitivity | F-QRDR-Hpylo | GCGTATTTTGTATGCGATGC |
|  |  | R-QRDR-Hpylo | ACAAAATCAATGGTGTCTTTATCA |
| *atpA* | MLST | atpA-for2^(f)^ | GGACTAGCGTTAAACGCACG |
|  |  | atpA-rev2^(f)^ | CTTGAAACCGACAAGCCCAC |
| *efp* | MLST | efp-for1^(f)^ | GGCAATTTGGATGAGCGAGCTC |
|  |  | efp-rev1^(f)^ | CTTCACCTTTTCAAGATACTC |
| *mutY* | MLST | mutY-for2^(f)^ | GTGGTTGTAGYTGGAAACTTTACAC |
|  |  | mutY-rev3^(f)^ | CTTAAGCGTGTGTYTTTCTAGG |
| *ppa* | MLST | ppa-for5^(f)^ | GGAGATTGCAATGAATTTAGA |
|  |  | ppa-rev5^(f)^ | GTGGGGTTAARATCGTTAAATTG |
| *trpC* | MLST | HptrpC6-for^(f)^ | TAGAATGCAAAAAAGCATCGCCCTC |
|  |  | HptrpC7-rev^(f)^ | TAAGCCCGCACACTTTATTTTCGCC |
| *ureI* | MLST | ureI-for^(f)^ | AGGTTATTCGTAAGGTGCG |
|  |  | ureI-rev2^(f)^ | GTTTAAATCCCTTAGATTGCC |
| *yphC* | MLST | yphC-for2^(f)^ | CACGCCTATTTTTTTGACTAAAAAC |
|  |  | yphC-rev3^(f)^ | CATTYACCCTCCCAATGATGC |

f. Primers presented on the PubMLST.org website. (Jolley et al., 2018)

Jolley, K. A., Bray, J. E., & Maiden, M. C. J. (2018). Open-access bacterial population genomics: BIGSdb software, the PubMLST.org website and their applications. *Wellcome Open Research*, *3*, 124. https://doi.org/10.12688/wellcomeopenres.14826.1
